# Effect of nutrient omissions on bread wheat and tef crops grain yield in Western Amhara, Ethiopia

**DOI:** 10.1371/journal.pone.0335174

**Published:** 2025-10-30

**Authors:** Beamlaku Alemayehu, Zerfu Bazie, Tadele Amare, Erkihun Alemu, Tarekegn Yibabie, Abere Tenagne, Atakltie Abebe, Abreham Awoke, Zelalem Addis, Zelalem Ayalneh, Tesfaye Feyisa, Getachew Agegnehu

**Affiliations:** 1 Adet Agricultural Research Center, Adet, Ethiopia; 2 Fenote-Selam Research sub-center, Fenote-Selam, Ethiopia; 3 Amhara Regional Agricultural Research Institute, Bahir Dar, Ethiopia; 4 International Crops Research Institute for the Semi-Arid Tropics (ICRISAT), Addis Ababa, Ethiopia; ICAR-Indian Institute of Rapeseed-Mustard Research, INDIA

## Abstract

The decline in soil nutrients in Ethiopia, particularly in Western Amhara, is causing low crop productivity. Some researchers have argued that the application of K, S, Zn, and B in blended, individual, and complex forms affects crop yield. Identification of the prime yield-limiting nutrient is the key to solvesoil nutrient problems. A field experiment was conducted at Burie-Wemeberema, Debere Elias, Gozamen and Gonji Qolela districts of Western Amhara in the 2022 cropping season. A composite soil sample was taken at a depth of 0–20 cm to determine soil chemical properties. Bread wheat and tef were used as a test crop. The gross plot sizes were 4m x 3m and the spacing between blocks and rows was 1.0 and 0.2 m, respectively. The experiment was laid out in a randomized complete block design with three replications and comprised of nine treatments: control, NPKSZnB-blended, NPKZnB, NPKSB, NPKSZnB, NPSZnB, NP, NPKSZnB-individually applied, and NPSZnB-compound+K. R programming software version 4.2.2 was used for data analysis, and treatment means were separated at P < 0.05 using the LSD test. The analysis of variance results showed that nitrogen and phosphorus are the most yield-limiting nutrients so far in the study area. Besides, omissions of potassium, sulfur, zinc, and boron did not show a significant (P < 0.05) effect on bread wheat and tef grain yield reduction as compared to the applied recommended nitrogen and phosphorus at all landscape positions of all study sites. Blended and compound nutrients also didn’t show a significant grain yield advantage as compared to the applied NP nutrients. Applied potassium, sulfur, zinc, and boron nutrients in blended, individual, and compound forms did not increase wheat and tef grain and biomass yields in all study areas. Currently, additions of K, S, Zn, and B nutrients in the fertilizer package do not have a significant grain yield advantage as compared to the recommended NP nutrients. We believe the present information on fertilizers in blended, compound, and individual forms is insufficient to draw any concrete conclusions. Therefore, we suggested further research to confirm which form of fertilizer and nutrient source is better for future crop production.

## 1. Introduction

More than 80% of Ethiopia’s population is dependent on agriculture, which contributes 50% to the country’s gross domestic product (GDP) and 80% to its export earnings [1]. Continuous crop production using high-yielding varieties, erosion and inadequate replacement of essential nutrients cause serious soil nutrient depletion [2]. Plant nutrition is the key critical factor controlling crop yield [3]. Consequently, this leads to a deficiency of essential plant nutrients. The deficiency of essential nutrient elements has been implicated in limiting the uptake of nutrients, growth, and yield of crops. The absence of macro and micro plant nutrients in the soil reduces crop yield. Deficiency of nitrogen and phosphorus significantly causes wheat and tef grain yield loss and their applications increase crop yield [4–8]. Research findings also indicated that the application of potassium, sulfur, and boron nutrients also increases crop yield [9,10].

Tef [*Eragrostis tef* (*Zucc*.) Trotter] is one of the most important gluten-free staple food crops in Ethiopia and has grown widely in the country for human consumption [11]. Tef has high nutritional content and is considered a protein source [12,13]. Despite its productivity is low as compared to the increased human population and demand in Ethiopia. Its national productivity is less than 1.8 t ha^-1^ [14]. (Wheat (*Triticum aestivum* L.) is also one of the most important cereal crop contributing to ensuring world food security [15]. Wheat is the third cereal crop produced after maize and rice in the world [15]. In Ethiopia, wheat is the third major cereal crop after tef and maize [14].

Earlier studies on soil and plant tissue analysis indicated that nitrogen (N), phosphorus (P), potassium (K), sulfur (S) and micronutrients like zinc (Zn) and boron (B) become deficient in Ethiopian soils [16–18]. In addition, K and S were also reported as a yield-limiting nutrient in wheat and rice crop production, respectively [19,20]. Recent studies on micro and macronutrients conversely indicated that nitrogen and phosphorus are the most yield-limiting nutrients of crop production [4–7,20–22]. Potassium, Sulfur, Zinc, and Boron were reported as not a yield-limiting nutrient of crop production in soils of Northwestern Ethiopia [4,5,7,21]. Application of N and P nutrients increased the yield, while K, S, Zn, and B nutrients applications did not significantly increase the yield [4–7]. Asfaw *et al*. [21] also reported that macro and micronutrients like K, S, Zn and B are not yield-limiting nutrients and nutrient management priority should be towards N and P nutrients. However, some researchers argued that in addition to N and P, applications of K, S, Zn and B affect the yield of crops [9,10,23–26]. In Ethiopia, the current research information on crop yield responses to different nutrient forms and rates is not sufficient. Accordingly, a nutrient omission research was conducted to test the hypothesis that applying different nutrient rates and sources would improve wheat and tef yield in different landscape positions. Therefore, this study was conducted to identify yield-limiting nutrients under different landscape positions for wheat and tef production.

## 2. Materials and methods

### 2.1. Description of the study area

The study was conducted on Foot, Mid, and hill slope landscape positions of most wheat and tef producing areas. The landscapes were selected based on the slope and elevation of the area. The experiment was conducted on three major wheat-growing potential districts of western Amhara: Burie-Wemeberema, Debere Elias, and Gozamen within the geographical coordinates 10° 16’ 56“- 10° 40’ 52” N latitudes and 36° 00’ 1” – 37° 36’ 15” E longitudes. The elevation of Bure Wemberema district study sites found between 2036–2107 m, DebreElias 2194–2231 m and Gozamen 2209–2286 m above sea level. The tef experiment was conducted in Gonji Qolela district with geographical coordinates 11° 12’ 43” N – 11° 13’ 57” N latitudes and 37° 34’ 36” E – 37° 37’ 16” E longitudes. The elevation of Gonji Qolela study sites was found between 2282–2340 m above sea level. The research work was conducted with the permission and coordination of Amhara Regional Agricultural Research Institute (ARARI, Bahir Dar, Ethiopia) and International Crops Research Institute for the Semi-Arid Tropics (ICRISAT, Addis Ababa, Ethiopia). Before planting, soil analysis results indicated that the cation exchange capacity (CEC in cmol^+^ kg^-1^ soil) was medium to high (19.98 to 45.80), pH (H_2_O) of the soil was strong to slightly acidic (5.28 to 6.40), available phosphorus (Ava. P in mg kg^-1^) in the soil was low to medium (4.80 to 10.00), total nitrogen (TN in %) was low (0.10 to 0.29), and soil organic carbon (SOC in %) was very low to low (0.70 to 2.54) in all landscape positions of the study areas.

### 2.2. Experimental design

The experiment comprised nine treatments, namely NPKSZnB-blended (All 1), NPKZnB (All-S), NPKSB (All-Zn), NPKSZn (All-B), NPSZnB (All-K), NP, NPKSZnB-individually applied (All 2), NPSZnB-copound+K (All 3) and the control (without nutrients). All 1, All 2, and All 3 have the same nutrient contents but were applied in blended, individual, and compound forms. Urea, DAP, KCl, NPS, ZnSO_4,_ and solubor (Borax decahydrate) (Na_2_B_4_O_7_.10H_2_O) were used as sources of nutrients for nitrogen, phosphorus, potassium, sulfur, zinc and boron, respectively. Fertilizer blending was done based on International Fertilizer Development Center (IFDC) guidelines using a small cement mixer at Debre Zeit Agricultural Research Center, Ethiopia. Nitrogen was applied in three splits for wheat (at planting, tillering, and booting stages) and in two splits for tef (at planting and tillering), while all other nutrients were applied at planting. The experiment was laid out in a randomized complete block design with three replications. Bread wheat (TAY variety) and Tef (Quncho variety) were used as a test crop. The gross and net plot sizes were 4m x 3m (12 m^2^) and 3.2m x 3m (9.6 m^2^), respectively, for both crops. Wheat crop was grown on 16 study sites, while tef crop was grown on 3 study sites. The spacing between blocks and rows were 1.0 and 0.2 m, respectively, for both test crops. The treatment setup is shown in Table 1.

**Table 1:** Treatment setup.

Treatments	Nutrient rates applied kg ha^-1^
All 1	N 120; P_2_O_5_ 76; K 60; S 14.8; Zn 1.5 and B 0.5
All – S	N 120; P_2_O_5_ 76; K 60; Zn1.5 and B 0.5
All – Zn	N 120; P_2_O_5_ 76; K 60; S 14.8 and B 0.5
All – B	N 120; P_2_O_5_ 76; K 60; S 14.8; and Zn 1.5
All – K	N 120; P_2_O_5_ 76; S 14.8; Zn 1.5 and B 0.5
NP	N 120 and P_2_O_5_ 76
All 2	N 120; P_2_O_5_ 76; K 60; S 14.8; Zn 1.5 and B 0.5
All 3	N 120; P_2_O_5_ 76; K 60; S 14.8; Zn 1.5 and B 0.5
Control	Without nutrients

### 2.3. Soil and agronomic data collections

#### 2.3.1. Soil sampling and analysis.

Before planting, one composite sample at 0–20 cm soil depth was collected to determine selected soil chemical properties. All collected soil samples were air-dried and crushed to pass through a 2-mm sieve. The selected soil parameters: soil pH-H_2_O, Ava. P, TN, SOC, and CEC were analyzed at the Adet Agricultural Research Center’s soil laboratory. Soil reaction (pH) was measured in 1:2.5 soil-to-water suspensions following the procedure used by [27]. Soil organic carbon (SOC) content was measured by the wet digestion method using the Walkley and Black method [28]. The total nitrogen was estimated using the Kjeldahl method [29], while the available phosphorus was analyzed by following the Olsen method [30]. The ammonium acetate extraction procedures were used to determine the soil cation exchange capacity [31].

The soil pH (H_2_O) was slightly acidic at the foot slope and strongly acidic at the mid and hill slope positions of Burie-Womeberema. Similarly, the pH of the soil was under a strongly acidic range at all landscape positions of Debre Elias, while at Gozamen, it was in a slightly acidic range at all landscape positions. The soil OC (%) was very low at all landscape positions of Gozamen and the foot slope landscape position of Debre Elias. The soil TN (%) was in the low range at all landscape positions, which was below the international critical standards, the Ava. P (mg kg^-1^) was low to medium at all landscape positions, while the CEC was medium to high at all landscape positions of the study areas. The soil OC, TN, and Ava. P was numerically high at the Foot and mid slopes as compared to the hill slope landscape positions of Burie-Womeberema and Gozamen and the mid-slope of Debre Elias study sites (Table 2). This result agreed with the findings of Amede *et al*. [32], who reported soil at the foot and mid-slope had higher amounts of organic carbon, TN, and Ava. P as compared to hill slopes.

**Table 2:** Selected soil chemical properties of Burie-Wemeberema, Debre Elias, Gozamen and Gonji Qolela.

Districts	Landscape positions	Descriptions	Parameters				
			pH (H_2_O)	SOC (%)	TN (%)	Ava. P (ppm)	CEC (meq/100g)
Burie & Wemberma	Foot slope	(one site)	5.69	2.6	0.19	7.33	28.48
	Mid slope	Range	5.28-5.49	2.13-2.33	0.19-0.21	9.27-9.53	23.2-26.04
		Mean	5.30	2.30	0.19	9.01	24.42
	Hill slope	Mean	5.44	2.31	0.18	6.79	27.44
Debre Elias	Foot slope	Range	5.30-5.30	1.79-2.07	0.18-0.21	4.87-7.75	32.00-33.20
		Mean	5.30	1.93	0.20	6.31	32.61
	Mid slope	(one site1)	5.47	2.54	0.24	8.74	19.98
	Hill slope	(one site)	5.35	2.46	0.24	6.23	24.98
Gozamen	Foot slope	(one site)	5.60	1.61	0.15	8.38	21.21
	Mid slope	Range	5.63-5.68	1.32-1.52	0.17-0.29	6.39-6.65	23.84-26.04
		Mean	5.66	1.42	0.23	6.52	24.94
		Range	5.78-5.85	0.92-1.74	0.14-0.16	4.92-12.3	27.12-27.32
	Hill slope	Mean	5.82	1.33	0.15	8.61	27.22
Gonji Qolela	Foot slope	(one site)	5.3	1.0	0.1	9.4	41.1
	Mid slope	(one site)	6.1	0.7	0.1	4.8	45.2
	Hill slope	(one site)	6.4	0.8	0.1	7.2	45.8
Critical limits			5.50	2.00	0.20	10.00	15.00
Ratings			Strongly to Slightly acidic	Very low to Low	Low	Low to Medium	Medium to High
Ratings are based on			Tadese [34]	Landon [35]	Metson [36]	Olsen [37]	Metson [36]

Where, pH = power of hydrogen, Ava. P = Available phosphorus (mg kg^-1^), TN = Total nitrogen (%), SOC = Soil Organic carbon (%), CEC = Cation exchange capacity (c mol^+^ kg^-1^ soil).

#### 2.3.2. Agronomic data.

For both test crops, total aboveground biomass and grain yield were measured. Harvesting was done from a 9.6 m^2^ net plot area (3.2 m by 3 m), with the outside rows left as buffers to prevent border effects. Plants harvested from the net plot area were sun-dried to a constant weight and converted to kg per hectare for statistical analysis. The grain yield was also calculated after threshing the biomass harvested from the net plot area and converted into kilograms per hectare. We adjusted the grain yield to 12.5% moisture.

### 2.4. Statistical data analysis

After homogeneity and normality tests were done, the essential agronomic data collected from field experiments for each parameter were subjected to analysis of variance (ANOVA) using R programming software. Treatment means were separated based on the least significant difference (LSD) test at P ≤ 0.05.

### 2.5. Partial budget analysis

A partial budget analysis was performed to investigate the economic feasibility of nutrient applications for bread wheat production. The output data (grain yield) were collected during threshing time and the input data (market price for applied fertilizers) were collected during the experimental year (2022) and used for analysis. The average grain yield of bread wheat was adjusted downwards by 10% to perfect the difference between the experimental plots’ yield and the yield farmers would expect from the same treatment under their own management.

## 3. Results

### 3.1. Response of wheat and tef grain and biomass yields to nutrients

**Fig 1:**
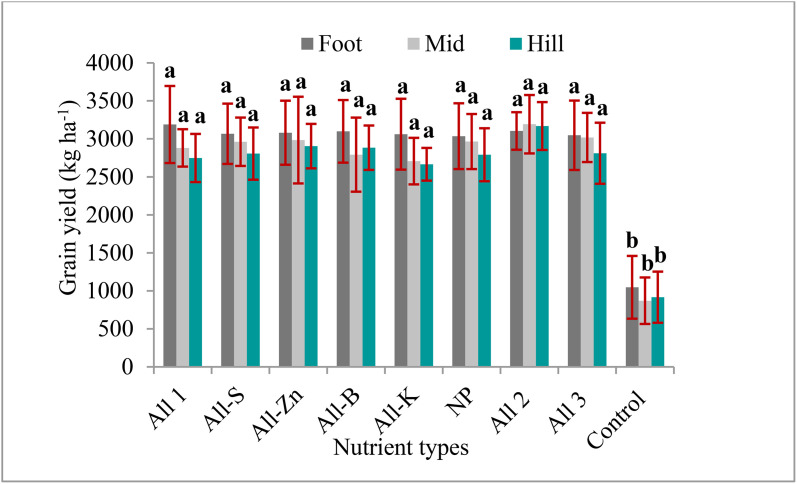
Combined wheat grain yield response to the applied nutrients across landscape positions in the study area. All1 = NPKSZnB-blended, All2 = NPKSZnB-individually applied, All3 = NPSZnB-compound+K, NP = All-KSZnB.

The analysis of variance (ANOVA) results showed that applying nutrients at all study sites resulted in a highly significant increase in wheat and tef grain and biomass yield as compared to the treatment in which no fertilizer was applied (Tables 3–6). The ANOVA results showed that potassium (K), sulfur (S), Zinc (Zn) and boron (B) nutrient omissions and or applications did not significantly affect the grain and biomass yield of wheat and tef in all landscape positions of all study areas. The ANOVA results revealed that there was no statistically significant bread wheat and tef grain and biomass yield observed among the treatments except the control in the landscape positions of the study areas. Omissions of K, S, Zn, and B nutrients did not show statistically significant bread wheat and tef grain and biomass yield reductions as compared to the applied recommended NP nutrients (Tables 3–6 and Fig 1).

**Table 3:** Effect of nutrient omission on wheat grain and biomass yield (kg ha^-1^) at Burie-Wemeberema.

	Landscape positions					
Treatments	Foot slope		Mid slope		Hill slope	
	GY	BY	GY	BY	GY	BY
All 1	3984a	10677a	2781a	7854a	3382a	7752a
All-S	3708a	10517a	2613a	7474a	3493a	7856a
All-Zn	3810a	10278a	3312a	8097a	3484a	7951a
All-B	3740a	10309a	2758a	9512a	3458a	7946a
All-K	3773a	9875a	2582a	7441a	3063a	7316a
NP	3668a	10035a	3273a	8462a	3481a	8102a
All 2	3277a	9042a	3331a	8336a	3789a	8766a
All 3	3733a	10340a	2786a	7447a	3582a	8476a
Control	1810b	5156b	1177b	2929b	1549b	3644b
LSD (0.05)	940	2402	1173	3024	1122	2414
CV (%)	15	15	37	34	29	28
Pr.	**	**	*	*	*	*

All1 = NPKSZnB-blended, All2 = NPKSZnB-individually applied, All3 = NPSZnB- compound+K, NP = All-KSZnB, GY = Grain yield. BY= Biomass yield.

*Means are significantly different at P ≤ 0.05.

**Means are significantly different at P ≤ 0.01.

**Table 4:** Effect of nutrient omission on wheat grain and biomass yield (kg ha^-1^) at Debre-Elias district.

	Landscape positions					
Treatments	Foot slope		Mid slope		Hill slope	
	GY	BY	GY	BY	GY	BY
All 1	3336a	8866ab	2514a	6809a	2428a	7219a
All-S	3151a	8502ab	2676a	7014a	2481a	7389a
All-Zn	3081a	8212b	1874a	5361a	2546a	7177a
All-B	3226a	8009b	1966a	6052a	2688a	7778a
All-K	3228a	8560ab	2251a	6219a	2610a	7233a
NP	3231a	8220b	2243a	6215a	2360a	6816a
All 2	3418a	9575a	2469a	7108a	2764a	7760a
All 3	3227a	9016ab	2612a	7372a	2230a	6434a
Control	939b	2655c	259b	878b	401b	1042b
LSD (0.05)	467	1132	1083	2201	796	1450
CV (%)	13	12	30	22	20	13
Pr.	**	**	**	**	**	*

All1 = NPKSZnB-blended, All2 = NPKSZnB-individually applied, All3 = NPSZnB- compound+K, NP = All-KSZnB, GY = Grain yield. BY= Biomass yield.

*Means are significantly different at P ≤ 0.05.

**Means are significantly different at P ≤ 0.01.

**Table 5:** Effect of nutrient omission on wheat grain and biomass yield (kg ha^-1^) at Gozamen district.

	Landscape positions					
Treatments	Foot slope		Mid slope		Hill slope	
	GY	BY	GY	BY	GY	BY
All 1	2247a	6434a	3348a	6519ab	2435a	7042b
All-S	2342a	6564a	3597a	6288ab	2445a	7043b
All-Zn	2351a	6757a	3766a	6470ab	2684a	7483ab
All-B	2331a	6276a	3653a	7082ab	2505a	7502ab
All-K	2184a	6304a	3289a	6398ab	2324a	7003b
NP	2208a	6434a	3378a	6337ab	2532a	7370ab
All 2	2617a	7040a	3778a	8311a	2953a	8894a
All 3	2182a	6304a	3658a	6184ab	2620a	7266ab
Control	392b	1250b	1176b	906c	800b	2092c
LSD (0.05)	446	1323	807	1755	756	1659
CV (%)	18	19	31	25	30	24
Pr.	**	**	**	**	**	**

Where, All1 = NPKSZnB-blended, All2 = NPKSZnB-individually applied, All3 = NPSZnB-compound+K, NP = All-KSZnB, GY = Grain yield. BY= Biomass yield.

**Means are significantly different at P ≤ 0.01.

**Table 6:** Effect of nutrient omission on Tef grain and biomass yield (kg ha^-1^) at Gonji Qolela district.

	Landscape positions					
Treatments	Foot slope		Mid slope		Hill slope	
	GY	BY	GY	BY	GY	BY
All 1	1467a	5111a	2032a	6638a	1655a	5496a
All-Zn	1341a	5201a	2145a	6496a	1497a	4860a
All-B	1454a	4917a	2239a	7399a	1457a	4715a
All-K	1329a	4975a	2041a	6599a	1551a	5613a
NP	1407a	5078a	2167a	6782a	1568a	5443a
All 2	1256a	4642a	1758a	6068a	1436a	4671a
All 3	1381a	5007a	2006a	5904a	1663a	5847a
Control	409b	1300b	711b	1876b	424b	1328b
LSD (0.05)	334	1048	491	1942	479	1554
CV (%)	15	13	15	19	19	19
Pr.	**	**	**	**	**	**

All1 = NPKSZnB-blended, All2 = NPKSZnB-individually applied, All3 = NPSZnB- compound+K, NP = All-KSZnB, GY = Grain yield. BY= Biomass yield.

**Means are significantly different at P ≤ 0.01.

Application of K, S, Zn, and B nutrients in blending, compound, and individual forms with N and P nutrients didn’t affect wheat and tef grain and biomass yields in all landscape positions of all study areas as compared to the applied recommended NP nutrients (Tables 3–6). The lower wheat biomass yield (878 kg ha^-1^) was recorded from the control treatment at the mid-slope landscape position of the Debre Elias district. Maximum wheat biomass yield (10677 kg ha^-1^) was recorded from the foot slope at Burie Wemeberma district. Lower wheat grain yield (259 kg ha^-1^) was recorded from the control treatment at mid-slope landscape positions of the Debre Elias district. Whereas the maximum wheat grain yields (3984 kg ha^-1^) were recorded from the foot slope at the Burie-Wemberema district. The lower tef grain yield (409 kg ha^-1^) was recorded from the foot slope landscape positions of Gonji Qolela district. Maximum tef grain and biomass yield (2239 and 7399 kg ha^-1^) were recorded from the mid-slope at Gonji Qolela district, respectively (Tables 3–6 and Fig 1).

The lower grain yields of wheat and tef crops were recorded from the control in all landscape positions and study areas (Tables 3–6). The lower yield of the control (unfertilized) indicates that the soil nitrogen and phosphorus nutrient supply status is insufficient to provide nutrients in optimum amounts (Table 2). This indicates that the current status of N and P applications is critically important. Our results showed that nitrogen and phosphorus nutrients caused significant wheat and tef yield reduction from the control treatment, since these nutrients increased yields with the addition of these nutrients.

**Fig 2:**
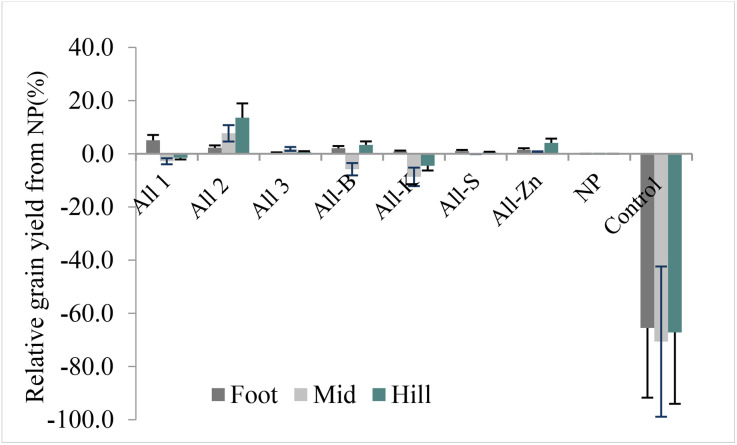
Bread wheat grain yield penalty from omitted nutrients relative to the applied recommended NP. All1 = NPKSZnB-blended, All2 = NPKSZnB-individually applied, All3 = NPSZnB-compound+K, NP = All-KSZnB.

We also compared treatments to determine how much yield was lost due to nutrient omissions across landscape positions. Due to nutrient omission, yield reductions ranged from 5 to 71% compared to the control treatment in the landscape positions of all study areas (Fig 2). The omission of K reduced wheat grain yield by 4.5% and 8.7% compared to the applied NP at the hill and mid-slope landscape positions, respectively. The omission of B reduced the yield by 5.8% at the mid-slope landscape position. A yield penalty of 65%, 71% and 67% was observed for the control treatment at the foot, mid and hill slope landscape positions, respectively (Fig 2).

### 3.2. Response of wheat plant height and spike length to nutrients

The omission of K, S, Zn, and B nutrients didn’t affect the vegetative growth of bread wheat during experimentation; almost all of the experimental pots showed similar vegetative performance. The ANOVA results showed that a highly significant increase in wheat plant height and spike length was observed, due to the applied nutrients compared to the control treatment (Table 7). These results revealed that omissions of K, S, Zn, and B nutrients did not show statistically significant wheat plant height and spike length reductions compared to the applied recommended NP. The lower wheat plant height (65.8 cm) and spike length (6.0 cm) were recorded from the control treatment at the mid-slope landscape position. Whereas the maximum plant height (102.8 cm) and spike length (8.9 cm) were recorded at the foot slope landscape position of the study area (Table 7).

**Table 7:** Effect of nutrient omission on wheat plant height and spike length (cm) of all study sites.

	Landscape positions					
Treatments	Foot slope		Mid slope		Hill slope	
	PH	SL	PH	SL	PH	SL
All1	101.9a	8.9a	98.2a	8.1ab	99.3a	8.1ab
All-S	101.3a	8.9a	98.7a	8.0ab	98.9a	8.3a
All-Zn	101.2a	8.7a	96.4a	7.7b	99.6a	8.2ab
All-B	99.8a	8.9a	95.7a	8.0ab	100.8a	8.1ab
All-K	101.0a	8.6a	96.2a	7.8ab	99.0a	8.1ab
NP	102.1a	8.8a	98.0a	8.1ab	98.7a	7.8b
All 2	102.3a	8.5a	99.4a	8.3a	100.1a	8.1ab
All 3	102.8a	8.6a	99.0a	8.2a	98.8a	8.0ab
Control	75.1b	7.4b	65.8b	6.0c	75.2b	6.9c
LSD (0.05)	4.4	0.6	4.8	0.5	4.1	0.4
CV (%)	5.5	8.4	8.4	11.1	5.9	6.4
Pr.	**	**	**	**	**	**

All1 = NPKSZnB-blended, All2 = NPKSZnB-individually applied, All3 = NPSZnB- compound+K, NP = All-KSZnB, PH = Plant height, SL = Spike length.

**Means are significantly different at P ≤ 0.01.

## 4. Discussion

Omissions of K, S, Zn, and B nutrients did not show significant reductions in wheat and tef grain and biomass yields in all landscape positions of the study areas (Tables 3–6). This indicates that K, S, Zn, and B are not yield-limiting nutrients in the study areas. These results agreed with the previous studies applying K, S, Zn, and B nutrients in blended, compound, and individual forms, which did not show a significant increase in wheat crop yield [4]. This indicates that K, S, Zn, and B nutrients are not yield-limiting nutrients so far in most crop-producing areas of Western Amhara. In addition, Agegnehu *et al*. [4]. concluded that various wheat-growing areas in Ethiopia do not require K, S, Zn and B t o achieve high yields. These results were also in line with the findings of Amare *et al*. [7] and Alemayehu *et al*. [5], who reported that applying K, S, Zn, and B nutrients did not show a significant yield advantage over the applied recommended NP nutrients. Our results also confirm the earlier studies, K, S, Zn, and B nutrient applications did not bring a significant increase in wheat and tef grain yield [6].

These results also align with the findings of Asfaw *et al*. [21], who reported that for wheat production, currently, there is no need for supplemental application of fertilizers containing K, S, Zn and B nutrients. However, these results disagreed with the findings of Kumar *et al*. [22], who indicated that applying K, S, Zn, and B nutrients had a significant effect on rice and wheat grain yield, and the highest grain yield was recorded from the treatment that received all nutrients. These results also contradicted the findings of (Chala *et al*. [9] and Seifu *et al*. [10], who reported that applying K, S, and B nutrients containing fertilizers increased wheat and tef crop yield. These results also disagreed with the findings of Dargie *et al*. [24], who concluded that applying balanced K, S, Zn and B nutrients significantly increased wheat crop yield.

The lower plant height and spike length in the control treatment indicated that N and P are the most growth-limiting nutrients, as plant height and spike length increased when N and P nutrients were applied. These results agreed with the findings of Alemayehu *et al*. [5], who concluded that N and P are the most plant height and growth-limiting nutrients. Previous studies by Agegnehu *et al*. [4] and Bazie *et al*. [6] indicated wheat and tef yield and yield attributes were significantly limited by N and P nutrients.

### 4.1. Partial budget analysis

The partial budget analysis was done based on CIMMYT [33]. The financial (partial budget) analysis results showed that blended, compound or individual application of K, S, Zn and B nutrients is not economical compared to the recommended NP. In comparison to the control and other treatments, nitrogen and phosphorus nutrients gave a higher marginal rate of return (866%) and a net benefit of 95, 643 ETB ha^-1^. (Table 8). According to the partial budget analysis, applying N and P nutrients only is the most economical for our smallholder farmers. These results agree with Agegnehu *et al*. [4], Asfaw *et al*. [21] and Bazie *et al*. [6], who reported no need to supplement K, S, Zn, and B but they recommended only N and P nutrients for wheat and tef production. The trend in financial analysis for tef is similar to that for wheat.

**Table 8:** Partial budget analysis.

Treatments	GY (kg ha^-1^)	AJDGY (kg ha^-1^)	TVC (ETB ha^-1^)	GB (ETB ha^-1^)	NB (ETB ha^-1^)	MC (ETB ha^-1^)	Dominance	MB (ETB ha^-1^)	MRR (%)	Rank
Control	945	850	0	33162	3316					
NP	2930	2637	7216	102859	95643	7216		62481	866	1
All-K	2812	2530	7874	98686	90811	658	D			
All-S	2945	2651	8870	103373	94504	1654	D			
All-B	2925	2633	9054	102668	93613	1838	D			
All-Zn	2990	2691	9181	104941	95760	1965		118	6	
All 2	3155	2840	9254	110744	101490	2038		5730	281	2
All 1	2939	2646	9254	103175	93920	2038	D			
All 3	2959	2663	9254	103857	94603	2038	D			

All1 = NPKSZnB-blended, All2 = NPKSZnB-individually applied, All3 = NPSZnB- compound+K, NP = All-KSZnB, GY = Grain yield, ADJGY = Adjusted grain yield, TVC = Total variable cost, GB = Gross benefit, NB = Net benefit, MC = Marginal cost, MB = Marginal benefit and MRR = Marginal rate of return. *D = Dominated; * 1USD = 52 Ethiopian birr (ETB). The price of bread wheat grain was 39birr kg^−1^.

## 5. Conclusions and recommendations

Applications of potassium, sulfur, zinc, and boron nutrients in blended, individual, and compound forms did not show a significant effect on wheat and tef grain and biomass yield advantage compared to the recommended NP in the study areas. On the basis of crop response, omissions of potassium, sulfur, zinc, and boron nutrients did not show a significant wheat and tef yield reduction in all landscape positions of the study areas. This indicates nitrogen and phosphorus are the most wheat and tef yield-limiting nutrients in the study areas. Based on our results, applying K, S, Zn and B nutrients for wheat and tef production is not economical for our smallholder farmers. We believe the present information on fertilizers in blended, compound, and individual forms is insufficient to draw concrete conclusions. Therefore, we suggested further research to confirm which form of fertilizer and nutrient source is better for future crop production.
